# A Novel Cost-Effective Nanobody against Fumonisin B1 Contaminations: Efficacy Test in Dairy Milk and Chickens

**DOI:** 10.3390/toxins14120821

**Published:** 2022-11-23

**Authors:** Yi Chen, Guanggang Qu, Hongkun Quan, Yihui Wang, Changjiang Wang, Md Atiqul Haque, Cheng He

**Affiliations:** 1 Key Laboratory of Animal Epidemiology and Zoonoses of Ministry of Agriculture, College of Veterinary Medicine, China Agricultural University, Beijing 100019, China; 2 Shandong Binzhou Animal Science and Veterinary Medicine Academy, Binzhou 256600, China; 3 Department of Microbiology, Faculty of Veterinary and Animal Science, Hajee Mohammad Danesh Science and Technology University, Dinajpur 5200, Bangladesh

**Keywords:** fumonisin B1, nanobody, phage-display technology, detoxification, efficacy test

## Abstract

Background: Fumonisin B1 (FB1) is a secondary metabolite produced mainly by *Fusarium verticillioides* or *Fusarium proliferatum*. It poses a huge threat to the sustainable animal industry and human health as well via food chains (egg, meat and milk). Although *E. coli*-expressed nanobodies are documented for diagnostic applications, nanobodies remain elusive as FB1 detoxifiers in feed and food. Results: In the present study, the *E. coli*-expressed nanobody was assessed to remove FB1 in fresh milk, embryonated eggs and broilers. Firstly, 2 alpacas received intramuscularly FB1-adjuvanted BSA 6 times, and then the variable domain of the heavy-chain antibody (VHH) of fb1 genes were amplified to clone into the pCANTAB 5 E vector in order to generate a VHH-FB1 phage antibody display library, yielding 3.4 × 10^10^ capacity with 96.7% positivity. Afterwards, 5 anti-FB1 nanobodies were expressed and identified. Furthermore, maximal 43.2% FB1 was removed from milk by 1:2000 concentration of nanobody 5 (Nb5). Furthermore, SPF-embryonated eggs were inoculated into albumens with nanobody-treated FB1. The Nb5 group yielded an 83.3% hatching rate, higher body weight, lower gizzard ulceration and fewer FB1 residuals. In order to warrant the above results, 50 broilers aged 10 days were received orally with 20 ppm of FB1 for 20 days. At the same time, birds were fed orally with 50 μg of Nb5 or bivalent nanobody 11 (BiNb11). Finally, the Nb5 group showed a higher relative body weight gain and lower gastric ulcerations and fewer inflammations in the thymus and bursa. Conclusions: Based on the above evidence, the Nb5 nanobody may be considered as an additional FB1 detoxifier, contributing to FB1 decontamination.

## 1. Introduction

Mycotoxins are toxic byproducts of fungi that have the ability to cause cancer, mutagenesis, teratogenicity, cytotoxicity, nephrotoxicity, neurotoxicity, immunotoxicity, dermotoxicity, and estrogenic potential. Their pollution has raised worries about the safety of feed and food throughout the world [1,2,3]. More than 500 mycotoxins, notably, aflatoxins, ochratoxins, trichothecenes, fumonisins, zearalenone, patulin, citrinin, and ergot alkaloids, have been identified in recent years [4]. Fumonisins (FBs) are a group of hydrophilic mycotoxins produced largely by the fungi Fusarium (*verticillioides, proliferatum, moniliforme, anthophilum, dlamini, globosum, fujikuroi, napiforme, nygamai, oxysporum*) and Aspergillus (*awamori, niger*) [2,4,5]. They frequently contaminate corn (maize), corn-based foodstuffs, asparagus, sorghum, soybeans, rice, pineapple, banana, sugarcane, beer, and animal feed worldwide, with FB1 being the predominant (>70%) cause of toxicity and posing a potential threat to human health and animal production [2,5]. FB1 has diverse serious impacts on various organs (brain, lungs, liver, kidneys, etc.) [6]. For example, it has been linked to equine leukoencephalomalacia in horse, porcine pulmonary edema syndrome in pigs, liver cancer in rat, stunted growth, developmental disorder, and neural tube defect in Brown Tsaiya Duck embryos, negative structural bone modifications in young chickens, and decreased hatchability and gizzard ulceration in chicken progenies [5,7,8]. Epidemiological data revealed that FB1 pollution in the human diet had a certain correlation with the high incidence of esophageal cancer, primary liver cancer, neural tube defects (NTDs), growth problem, idiopathic congestive cardiopathy (ICC) and other diseases in humans [5,6,7,9]. Furthermore, FB1 has been listed as a possible Group 2B human carcinogen by the International Agency for Research on Cancer (IARC) [10], with a tolerable daily intake (TDI) of 2 µg/kg BW/day set by the joint Food and Agricultural Organization (FAO) [11] and World Health Organization (WHO) [11].

A recent study on female BALB/c mice revealed that FB1 could cause significant hepatotoxicity, nephrotoxicity, and hematological toxicity, indicating that the foregoing maximum TDI of FB1did not appear to provide adequate protection [1]. In lower doses, FB1 triggers cell death in all body parts of plants, both at the cellular and organ levels, and has similar adverse effects on animals and humans [2]. Confronting high FB1 contaminations in food chains, it is still a big challenge for scientists to innovate novel FB1 detoxifier. Therefore, attempts have been made for the discovery of an effective FB1 detoxification method. Physical, chemical, and biological methods of detoxification are currently available. Furthermore, both organic and inorganic mycotoxin binders have been used to limit toxicity in animal feed. Feed and food by-products contaminated with FB1 can be destroyed by heat (>150–200 °C) and alkali treatment to reduce FB1 damage to the body [12]. In vitro, tri-octahedral bentonites, an inorganic binder, have been shown to adsorb >90% of Zearalenon (ZEN) and FB1 [13]. Isothiocyanate (ITC), which contains electrophilic carbon and can react with the free amino groups of mycotoxins, reduced the levels of FB1 by 53–96%, FB2 by 29–91%, and FB3 by 29–96% through ITC fumigation. These results suggest that the primary amine group of FB is critical to its toxicity, as the naturally occurring acetylfumonisin B is not considered toxic. FB can also react with reducing sugars (glucose or fructose) to block primary amine groups and can undergo the Maillard reaction to form N-carboxymethyl-FB1, which is less toxic, and detoxify FB1 contamination [14,15]. Traditional physical and chemical methods can only achieve partial FB reduction while depleting nutrients and having undesired effects. With the development of modern biotechnology, metabolic detoxification has gradually supplanted physical and chemical detoxification as the primary method for mycotoxin reduction. Metabolic detoxification has mild action and does not reduce the nutritional value or the palatability of feed [4]. Some effective enzyme preparations and strains can reduce FB1 toxicity in feed. The two genes *fumD* and *fumI* of *Sphingopyxis* spp. MTA144 were found to produce enzymes that catalyze the de-esterification and deamination of FB1 in a sequential manner, detoxifying FB1 through their continuous action. The *Pseudomonas* genus was identified as a significant FB1 degradation member using 16 SrDNA sequencing and antibiotic-driven selection of a bacterial consortium (SAAS79) and its crude enzymes with strong FB1 degradation activity (90%) isolated from waste water mushroom [9]. Moreover, the hydrolase and transferase enzymes of Serratia marcescens are capable of degrading FB1 at a rate of 37% [15]. Although many studies have been focused on the degradation of FB, there is still a lack of effective methods for FB1 degradation in food and feed.

Nanobodies (Nbs), also known as single-domain antibodies, are derived from the variable domain of heavy-chain antibodies (VHH) in camels and are thought to be the smallest intact antigen-binding fragment currently available [8,9]. Nbs have many unique antibody characteristics, including small molecular weight (15 kDa), high solubility, high specificity, high affinity, strong stability, and easy cloning. Importantly, industrial production of recombinant nanobodies by microorganisms is highly cost-effective, and nanobodies can be easily utilized as building blocks for multi-domain constructs [16,17]. Nbs have high stability to organic solvents, which is useful for mycotoxin immunoassays. He et al. assessed Nbs’ solvent stability against AFB1 and found that it was more stable to methanol, acetone, and acetonitrile than monoclonal antibody (mAb) [18]. Kunz et al. examined the thermostabilities of 78 pure nanobody binders and determined that the stability of eight modified nanobodies varied by a mean of 2.3 °C and a maximum of 6.1 °C [19]. Alsulami et al. reported the FNanoBiT assay for the detection of FB1 in the maize extract, and the relative standard deviation (RSD) observed suggested high stability allowed it to be better suited for field application [6]. Due to the aforementioned advantages of nanobodies, their application in the detection of mycotoxins in agricultural products has gained increasingly attention in recent years. The anti-FB1-idiotypic nanobody and alkaline phosphatase were fused to express, and a one-step competitive enzyme-linked immunosorbent assay (ELISA) method was established for detecting FB1 level in grains. Likewise, for FB1 detection, a noncompetitive idiometric immunoassay using a combination of β-type anti-idiotypic Nb and phage-displayed α-type anti-idiotypic Nb revealed a 17-fold increase in sensitivity compared to the competitive ELISA (LOD = 3.41 ng/mL), implying that this approach has broad utility for checking small molecules in foods [17]. In addition to the ELISA detection method, the application of an immunosensor for aflatoxin B1 (AFB1) detection also been established by using an Nb. For instance, an Nb, conjugated by a horseradish peroxidase, is combined with a hybridization chain reaction signal amplification system to achieve rapid and ultra-sensitive AFB1 detection. Under ideal conditions, the LOD of the immunosensor was 68 fg/mL, and the linear range was 0.5–10 ng/mL with a sensitivity of 2.7 μA • (mL/ng) [20]. So far, scarce reports have been published on toxin neutralization with nanobodies. In 2016, Andersen et al. immunized llamas with toxin B and obtained four Nbs capable of neutralizing toxin B in an in vitro cell-based assay [21]. Further, the in vivo protective effect was validated in a hamster model. When challenged with *Clostridium difficile*, half of the hamsters survived after being treated with Nbs expressed in *Lactobacillus* and showed no damage or only limited inflammation of the intestinal mucosa. In another investigation, Harmsen et al. showed that oral administration of high doses of nanobodies against *Escherichia coli* F4 pili reduced *E. coli*-induced diarrhea in piglets [22]. However, no report has been published until now on the detoxifying effect of Nbs on FB1.

In this study, FB1-specific nanobodies were developed by phage-display technology and expressed in an *E. coli* expression system. The binding ability of the Nbs was analyzed by ELISA and their antagonistic effect on FB1 was verified via chicken embryo model and broiler model. Our results showed that Nb5 was identified to reduce FB1 toxicity on embryonated eggs and it also improved body weight gain and reduced gizzard ulceration in broilers, suggesting that the nanobody Nb5 had the effect of antagonizing FB1 and potential application for human food additive and poultry industry.

## 2. Results

### 2.1. Screening and Identification of FB1 Nanobodies

The alpaca was immunized with FB1-adjuvanted-BSA whole antigen. Post the final inoculation on day 4, the titers of the FB1 nanobody were detected to be 1:5000 in alpaca sera by an indirect ELISA. The lymphocytes were isolated from a total volume of 200 mL of alpaca peripheral anticoagulant. After extracting RNA, it was reversely transcribed into cDNA. The *VHH* fragment was amplified by nested PCR, and the target band of approximately 700 bp was recovered in the 1st round and the approximately 400 bp was produced in the 2nd round. The *VHH* fragment was inserted into a pCANTAB 5 E vector and electrically converted to TG1-competent cells to obtain a phage-display library. The library capacity was roughly 3.4 × 10^10^ and the positive rate was approximately 96.7%.

After the 3rd round of panning, the enrichment rate was identified to be 16. After the 3rd rounds of panning, the recombinant bacteriophage was infected into logarithmic TG1 cells, LB/AMP-Glu plates were coated, and 60 single colonies were randomly selected. The reactivity of the crude extract with FB1-OVA was identified by ELISA assay. Out of the 60 selected monoclonal clones chosen, 58 positive clones were sequenced and analyzed, where 5 nanobody phages coding different sequences were identified and named as Nb5, Nb11, Nb12, Nb13, and Nb16 in sequence.

### 2.2. Prokaryotic Expression and Identification of Anti-FB1 Nanobody

**Figure 1:**
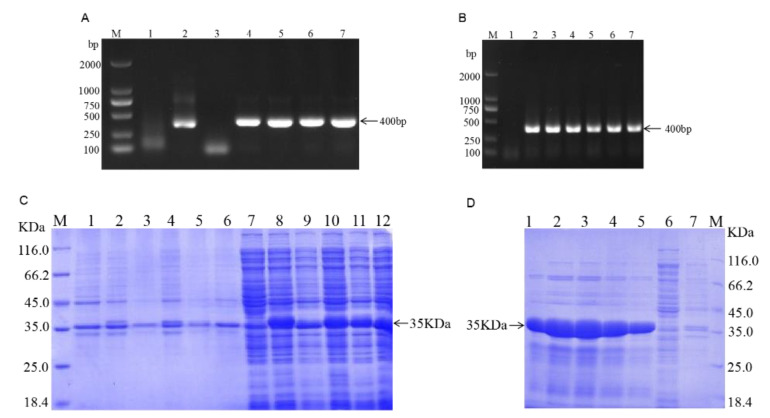
Construction of the *VHH*-FB1 prokaryotic expression vector and expression of VHH recombinant protein. (**A**) Cloning *VHH*-FB1 gene. Lane M, DNA marker (TAKARA); Lane 1, Negative control of *VHH*-FB1 gene (400 bp); Lane 2–7, PCR products of *VHH*-FB1 gene; (**B**) PCR colony. (**C**) Expression of VHH recombinant protein (M. Protein marker; 1. Precipitation of bacteria of PSF without induction; 2–6. Precipitation of bacteria of Nb1–Nb5 with induction; 7. Supernatants of bacteria of PSF without induction; 8–10. Supernatants of bacteria of Nb1–Nb5 with induction). (**D**) Purification of VHH recombinant protein (1–5. Recombinant protein eluted with 250 mM imidazole concentration; 6. Supernatant of flow-through; 7. Precipitation of flow-through).

The target fragment was amplified by specific primers and the PCR amplification produced a roughly 400 bp band, which was compatible with the expected target band size (Figure 1A). For PCR identification, a single colony was picked and incubated overnight at 37 °C. The prokaryotic expression vector of *VHH*-FB1-PSF was constructed successfully (Figure 1B). Afterwards, the *VHH*-FB1-PSF plasmid was transformed into *E. coli* BL21(DE3) competent cells, and IPTG was added to a final concentration of 1 mM before incubation overnight at 37 °C. The proteins were expressed as soluble proteins and then identified by SDS-PAGE (Figure 1C). The recombinant Nbs were confirmed to be approximately 35 KDa in size. The supernatants were purified with Ni-NTA columns at room temperature and then identified by SDS-PAGE (Figure 1D).

### 2.3. Nb5 Detoxification on FB1-Contaminated Milk

**Figure 2:**
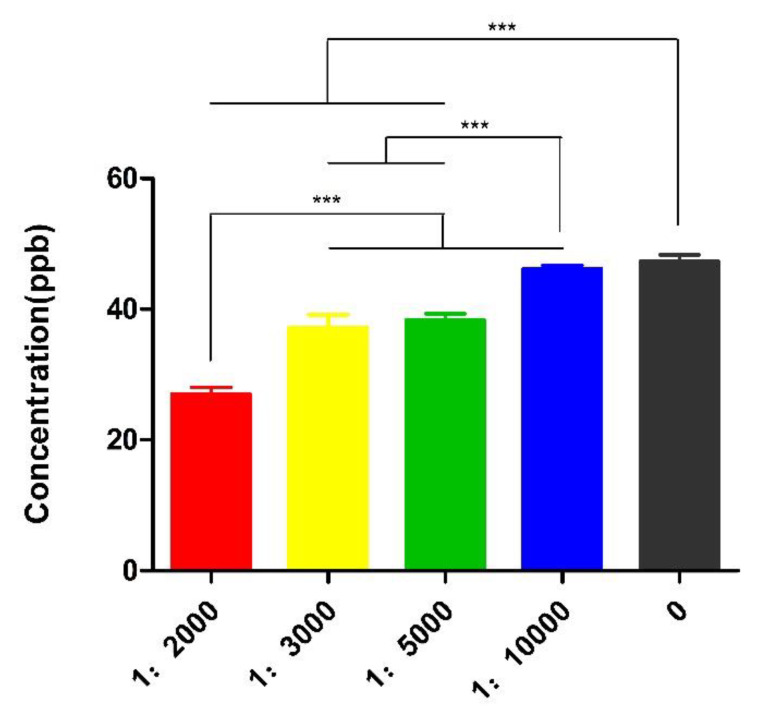
Detoxification of Nb5 at different concentrations. A dose-dependent manner of decontamination was found among 1:2000, 1:3000, 1:5000 and initial solutions post treatment at 25 °C for 2 h. The maximal detoxification was determined to be 1:2000 solution. The data were expressed as mean ± SD.; ***: *p* < 0.01.

Serial concentrations of Nb5 were incubated with FB1-contaminated milk sample at 25 °C and significant difference of toxin removal was determined post treatment with the initial solution, 1:2000, or 1:3000, or 1:5000, or 1:10,000, while a dose-dependent manner of detoxification was 43.3%, 21.98%, 19.62%, and 3.07%, respectively (*p* < 0.01). Obviously, the optimal detoxification was determined to be 1:2000 solution and FB1 concentration was reduced to 27.04 ppb with a degradation rate of 43.26% (Figure 2).

### 2.4. Effect of Nanobodies on Growth and Gizzard Ulceration of the Embryonated Eggs

**Figure 3:**
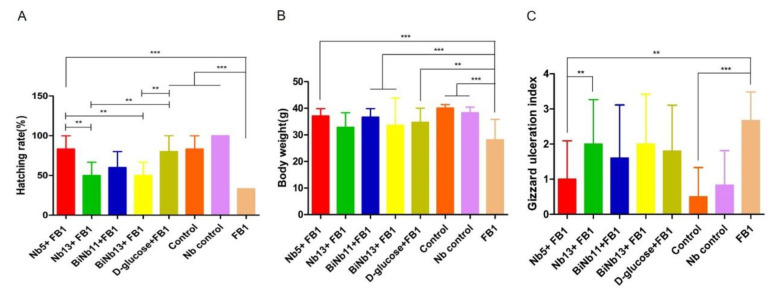
Effects of nanobodies on FB1-contaminated chicken embryos. (**A**): Hatching rate post inoculation with FB1 or in combination with monomer nanobodies, bivalent nanobodies, and D-glucose. Lower hatchability was observed in the FB1 control group compared to other groups (*p* < 0.01). Both the Nb5 + FB1 group and the D-glucose group yielded higher hatching abilities than other Nb groups did in the experiment. No statistical difference was determined between the Nb5 + FB1 group and the D-glucose + FB1 group either (*p* = 0.795). (**B**): Hatching body weight after treatment with monomer nanobodies, bivalent nanobodies, and D-glucose. Slower growth rate was observed in the FB1 control group compared to the other groups (*p* < 0.01). However, no statistical difference of body weight was found among the Nb5 + FB1 group, or the Nb13 + FB1, or the BiNb11 + FB1 group, or the BiNb13 + FB1 group, or the D-glucose + FB1 group (*p* > 0.05). (**C**): Lesion scores of gizzard ulceration of chicken post FB1 treatment with monomer nanobodies, bivalent nanobodies, and D-glucose. Postmortem, the FB1 group induced higher lesions of gizzard ulcerations compared to the Nb5 + FB1 group, the BiNb11 + FB1 group (*p* < 0.05), and the control group (*p* < 0.01). No significant difference was found in the Nb13 + FB1 group, BiNb13 + FB1 group, and the D-glucose + FB1 group (*p* > 0.05) and no significant difference was found between the Nb5 + FB1 group and the D-glucose + FB1 group either (*p* = 0.255). The data were expressed as mean ± SD. **: *p* < 0.05, ***: *p* < 0.01. Control: PBS; Nb control: Bi-Nb-11 nanobody.

On day 21, the hatchability of the embryonated eggs exposed to 64 μg of FB1 was significantly lower than that of the control groups or the nanobody-treated groups (*p* < 0.01). The hatchability was 83.33% and 80%, respectively, in the Nb5 + FB1 group and the D-glucose + FB1 group. However, lower hatchability was found in the Nb13 + FB1, the BiNb11 + FB1, and the BiNb13 + FB1 compared to the Nb5 + FB1 group (Figure 3A).

As for body weight of the new-borne chickens, the FB1 group induced slow growth compared to the control groups and the Nb control group (*p* < 0.01). However, no statistical difference was found among the Nb5 + FB1 group, or the BiNb11 + FB1 group, the BiNb13 + FB1 group and the D-glucose + FB1 group (Figure 3B). Postmortem, FB1 induced a highly gastric ulceration index while lower lesions were determined in the Nb5 + FB1 group (*p* < 0.05) and all the control groups (*p* < 0.01). In addition to the Nb5 + FB1 group, no statistical difference was found among the Nb13+FB1 group, the BiNb11 + FB1 group, the BiNb13 + FB1 group, and the D-glucose + FB1 group (Figure 3C).

**Figure 4:**
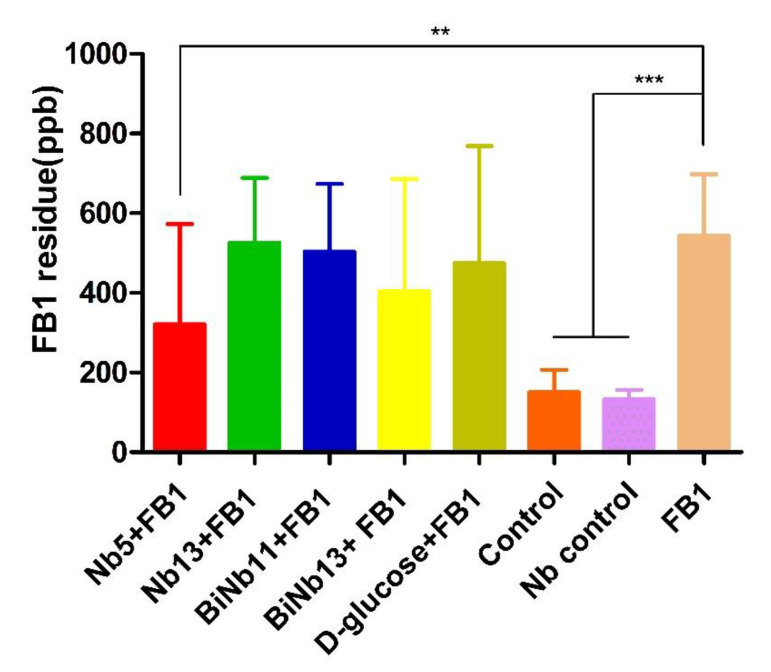
FB1 residuals in the lungs and gizzard of new-borne chickens. Lower FB1 contamination was observed in the Nb5 + FB1 group compared to that of the FB1 control (*p* < 0.05). However, no significant difference was found among the Nb13 + FB1 group, the BiNb11 + FB1 group, and the D-glucose + FB1 group (*p* > 0.05) and no significant difference was found between the Nb5 + FB1 group and the D-glucose + FB1 group either (*p* = 0.448). No residual FB1 was detected in the gizzards of all groups. The data were expressed as mean ± SD.**: *p* < 0.05; ***: *p* < 0.01. Note: Control: PBS; Nb control: Bi-Nb-11 nanobody.

As for FB1 residues in the lungs of hatching chickens, lower FB1 contamination was determined in the Nb5 + FB1 group and the BiNb13 + FB1 group compared to that of the FB1 control (*p* < 0.05). However, no significant difference was found among the Nb13 + FB1 group, the BiNb11 + FB1 group, and the D-glucose + FB1 group (*p* > 0.05). No residual FB1 was detected in the gizzards of all groups (Figure 4).

### 2.5. Effect of Nanobodies on Growth, Gizzard Ulceration, and Antibody Levels of the Broilers

**Figure 5:**
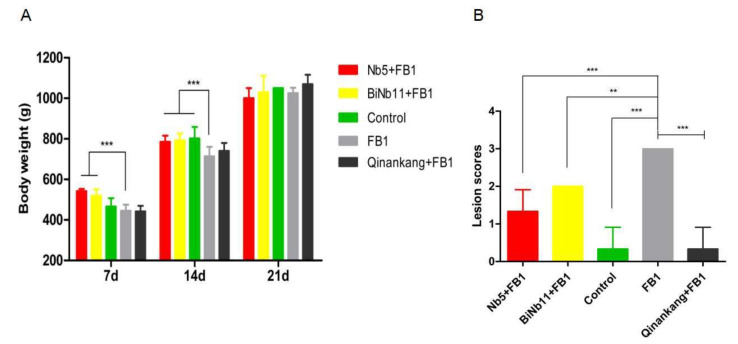
Effects of nanobodies on the FB1-contaminated broiler’s development. (**A**) Both Nb5 + FB1 and BiNb11 + FB1 improved body weight on day 7 and on day 14 compared to the FB1 control (*p* < 0.01). No discernible difference was found among all the groups on day 21 (*p* > 0.05). (**B**) Lesion scores of gizzard ulceration of broiler chickens post treatment with Nb5 and BiNb11. Post mortem observation, both the Nb5 + FB1 group (*p* < 0.01) and BiNb11 + FB1 (*p* < 0.05) group developed lower lesions of gizzard ulcerations compared to the FB1 control group. However, no statistical difference was found between the Nb5 + FB1 group and the commercial detoxifier, Qingankang (*p* > 0.05). The data were expressed as mean ± SD. **: *p* < 0.05; ***: *p* < 0.01.

Regarding the body weight of the broilers (Figure 5A), FB1 greatly reduced the body weight of broilers on day 7 and day 14 (*p* < 0.01). Significant increasing body weight was determined in the Nb5 + FB1 group and the BiNb11 + FB1 group in the first two weeks (*p* < 0.01). However, no significant difference was observed among all the groups in the final week (*p* > 0.05). Post-mortem, lesion score index was used to access the gizzard ulceration. Obviously, FB1 aggravated gizzard lesions in broilers and gastric ulceration index amounted to 3.0 ± 0.0. On the contrary, lower gizzard ulceration index was observed in the Nb5 + FB1 group (*p* < 0.01), the BiNb11 + FB1 group (*p* < 0.05), and the Qinankang + FB1 group (*p <* 0.01), compared to the FB1 group. However, no significant difference was found among the Nb5 + FB1 group, the BiNb11 + FB1 group, and the Qinankang + FB1 group (*p >* 0.05) (Figure 5B).

**Figure 6:**
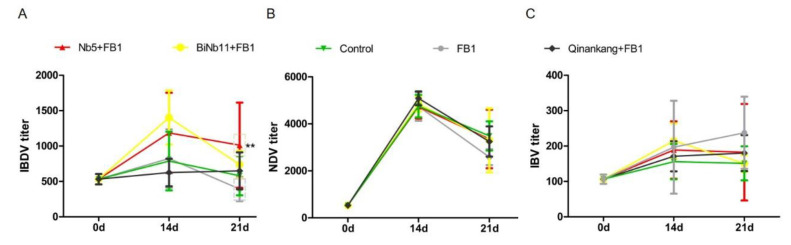
Effects of nanobodies on FB1-contaminated broiler’s humoral responses. (**A**): On day 14 and 21, the Nb5 + FB1 group induced higher IBDV antibody titers than the Control group or the Qinankang + FB1 group or the FB1 group (*p* < 0.05), whereas no significant difference was found between the Nb5 + FB1 group and the BiNb11 + FB1 group (*p* > 0.05) throughout the study. (**B**): On day 21, both the Nb5 + FB1 and BiNb11 + FB1 groups yielded higher NDV antibody levels than the FB1 control group, whereas no statistical difference was found on day 14 (*p* > 0.05). (**C**): Regarding the IBV antibody, both the Nb5 + FB1 and BiNb11 + FB1 groups yielded a low antibody compared to the FB1 control group, whereas no significant difference was determined among all the groups (*p* > 0.05). The data were expressed as mean ± SD. **: *p* < 0.05.

Post treatment, antibody titers against Newcastle disease virus (NDV), Infectious bronchitis virus (IBV), and Infectious bursal disease virus (IBDV) were determined from initial day to day 21. Increasing antibody titers against IBDV were determined in the Nb5 + FB1 group compared to other groups on day 14 and day 21 (*p* < 0.05); no significant difference was found between the Nb5 + FB1 group and the BiNb11 + FB1 group during all the observation (*p* > 0.05) (Figure 6A).

As for NDV antibody response, the increasing antibody levels were determined both in the Nb5 + FB1 group and the BiNb11 + FB1 group compared to the FB1 control on day 21, but no statistical difference was found in the study (*p* > 0.05) (Figure 6B). Regarding the IBV antibody, on day 21, compared to the FB1 control group, the IBV antibody level of the Nb5 + FB1, BiNb11 + FB1 and Qinankang groups were lower and moreover, the Nb5 + FB1 and BiNb11 + FB1 showed a declining trend of antibody level. However, there is no significant difference of IBV antibodies level among all groups (*p* > 0.05) (Figure 6C).

## 3. Discussion

In this study, five FB1 toxin-specific Nbs were obtained post immunization in the alpaca. Embryonated eggs are recommended as a toxicity bioassay due to FB1 and DON contaminations [23]. We used the embryonated eggs to test the efficacy of the five obtained nanobodies against FB1 embryo toxicity. The primary amino group of FB plays an important role in the toxicity of FB1, because N-acetyl-FB1 was not considered toxic. Lu et al. found that D- glucose reacted with the primary amino group of FB1 at 60 °C and induced its detoxification [15]. Our results showed that Nb5 not only reduced FB1 in the contaminated milk at 1:2000 dilution, but also increased egg hatching ability and body weight and reduced gizzard ulcerations of new-borne chickens. Moreover, the Nb5 and BiNb11 improved the broiler’s body weight and the Nb5 also reduced gizzard ulceration, which was comparable to the popular commercial detoxifier, Qiangankang, as the positive control. Our study revealed that Nb5 exerted decontamination by detoxifying FB1 toxins.

Regarding detoxification of mycotoxin contamination, physical measure and chemical decontamination techniques may be quite efficient. However, the more sustainable and restricted use of fungicides require new approaches to control this hazard. Food safety demands permanent research efforts for exploring new strategies to reduce mycotoxin contamination. In the present study, Nb5 was identified to remove the FB1 toxin in dairy milk, with a suggestion of a potential detoxifying agent due to an efficient and robust method for the generation of antibodies against a wide range of targets with highly specific binding properties. FB1 was documented to be transmitted from diet into animal milk and exerted toxic effects on human and animal health. The interaction of different mycotoxins may be additive or synergetic. Given that milk is a source of nutrients, especially in childhood, a thorough investigation of the occurrence of mycotoxins as well the adoption of measures to minimize their contamination of milk is urgently needed. Moreover, FB1 and FB2 in milk samples are stable to pasteurization (62 °C/30 min) and storage at 4 °C for 11 days [8], and the incidence of these contaminants is a major issue for human health. The carry-over of fumonisin B1 from contaminated feed into dairy milk also suggests its carry-over from contaminated food into breast milk in Northern Tanzania; 10.3% had fumonisin B1 levels above the EU limit of 200 ppb for fumonisins in infants’ food that leads to unacceptable exposures in infants [24]. In the present study, a 43.26% detoxifying rate was determined in fresh milk post treatment with 1:2000 concentration of Nb5 concentration. Compared to the anti-FB1 monoclonal antibody, a 25% degradation rate of FB1 was determined post incubation at 25 °C for 2 h in our previous study [25]. Regarding cost-effective detoxification, Nb5 might be a promising detoxifier for combating mycotoxin contamination in the daily milk consumption. The nanobody is the smallest specific binding entity compared to the monoclonal antibody and polyclonal antibody. In addition, the nanobody will avoid random chemical conjugation and the use of secondary antibodies, contributing to mycotoxin removal in the study.

Nanobodies have benefits over standard monoclonal and polyclonal antibodies in terms of size, stability, and expression level when used as antibodies. There have been some successful examples regarding the use of Nbs to effectively recognize small molecules, such as the mycotoxins AFB1, OTA, and 15-acetyl-DON, and some environmental contaminants. The isolated anti-idiotypic-Nb was subjected to an ELISA for the detection of FB1 contaminated in cereals and feedstuffs [26]. The developed assay showed an IC50 value of 0.95 ng/mL, with a limit of detection of 0.15 ng/mL, linear range of 0.27–5.92 ng/mL, and low cross-reactivity toward FB2 (4.93%). The sensitivity of anti-idiotypic ELISA was enhanced approximately 20-fold compared with that of the chemosynthetic FB1-BSA conjugates-based ELISA (IC50 = 21.14 ng/mL). The established anti-idiotypic ELISA was validated to be suitable for monitoring the total fumonisin concentration under the current regulatory limits of fumonisins in most countries [18]. In the present study, we tested the ELISA titers after the 4th immunization of alpaca; the titer reached only 1:100, which might be related to the low dose of the FB1 compound. Our phage-display library yielded a capacity of 3.4 × 10^10^ after identification, and 46 single colonies were randomly selected, with a positive rate of 96.7%. The library showed good diversity once the positive monoclonal antibodies were sequenced. The library was then subjected to rescue, including 3 rounds of selective panning and enrichment, and 5 nanobodies with different amino acid sequences were finally screened out.

The detoxification was further verified by embryonated eggs and the broiler model. A previous study confirmed that FB1 had moderate toxicity in chick embryos, causing pathological changes, such as hydrocephalus, beak enlargement, neck elongation, as well as heart, lung, liver, kidney, and small intestine abnormalities [27]. In a prior study, chick embryos were inoculated with 16 µg FB1 per embryo and examined for the presence of any serious developmental defects, characterized as severe hemorrhagical inflammations evident in the head, neck, and chest of the deceased embryo [28]. In a recent study, 24 µg FB1 was inoculated into 11-day-old chicken embryos, resulting in lung hemorrhage and gastric ulcer in the new-borne chickens, as well as FB1 residuals in gizzard and lung [8]. In the current study, the average body weight was significantly reduced, indicating that FB1 had certain effects on the development of chicken embryos. In the present study, Nb5 not only improved the egg hatching ability and body weight. It also alleviated the gizzard ulcers, while the D-glucose did not reduce the gizzard ulceration of new-born chickens. More interestingly, broilers’ gizzards health was improved greatly post Nb5 (*p* < 0.01) and the BiNb11 (*p* < 0.05) treatment, without a significant difference comparable to the treatment of the commercial detoxifier product, the Qingankang group (*p* > 0.05) (Figure 5B). In terms of clinical efficacy, Qinankang with 20% protocatechuic acid (PCA) is commercialized against mycotoxin contamination in the broiler industry, characterized by fewer gizzard ulcerations and enhanced body weight in the study (Figure 5A). However, no stimulating IgG responses against IBDV, NDV, and IBV were observed in the Qinankang group compared to those of the Nb5 + FB1 group. As for material supplying, both PCA and nanobody are environmentally friendly products using *E. coli*-engineered strains in the fermentation process. The nanobody is cheaper to produce for large scale production than PCA in the manufacturer due to complicated purification. In this sense, the nanobody might be a low-cost detoxifier in the poultry industry.

FB1 has diverse effects on the immune system, causing both stimulation and suppression of the response to foreign antigens, and apparently inducing an antigenic response to FB1 [29]. Our previous study indicated that the combination of FB1 and DON was associated with a low hatching rate and gizzard ulcerations in chicken progenies [8]. The previous report confirmed that FB1 was an immunosuppressive to chickens when present in the diet with a ratio of 200 mg FB1/kg [30]. In the present study, our data confirmed that limited immune suppression against IBDV was induced in the birds that received 10 mg FB1/kg of diet for 14 days. No significant suppressive impact on NDV and IBV was observed in the study. However, Nb5, as a monomer nanobody, could enhance the IBDV humoral response of chickens exposed to daily FB1 diet.

Nanobodies termed as VHH antibody are usually isolated from the library constructed by primary antibody immunization and eluted with the free antigen. Although the nanobodies are a particularly useful tool for monitoring mycotoxins in food and feedstuffs, as they are easily genetic engineered and have superior stability [31], the potential removal of mycotoxin has not been fully investigated. Nanobodies are reported to recognize the active site of the antigen, and also serve as a surrogate for the original antigen and compete with the original antigen for the primary antibody [32]. In the present study, both monomer VHH and bivalent VHH were produced in large quantities, excellent solubility, and resilience to gastric pH value. Post oral administration with Nb5 and BiNb11, nanobodies might bind to FB1 and block the specific site of the toxin, leading to an improvement of body weight and gastric lesions. Compared to the bivalent VHH of BiNb11, the monomer Nb5 showed the advantages of chicken health and embryo development, a suggestion of highly affinity to FB1 and efficient binding activities. Initially, bivalent and monomer nanobodies were used for toxin quantitative, and our presented data indicated novel detoxifiers in poultry industry and diary consumption. Further investigation is needed to verify the commercial administration. On the other hand, more understanding is needed about the conversion procedures, the toxicological characteristics of the products obtained by transformation, the effect of the conversion on the nutrition of feed and on animal safety. Such feed additive must be harmless and stable in the digestive tract of animals. Therefore, future work is required to elucidate wide application as feed or food detoxifier.

## 4. Conclusions

In the present study, 5 nanobodies that specifically bound to FB1 were successfully screened out using phage-display technology. Based on detoxifying efficacy in fresh milk, chicken embryos, and broilers, it is the first report in which the monomer nanobody termed Nb5 has been identified to be a putative novel detoxifier by degrading FB1 mycotoxins in fresh milk, embryonated chickens and broiler, contributing to sustainable poultry industry and food safety for human beings.

## 5. Materials and Methods

### 5.1. Alpaca Immunization

Two adult male alpaca received subcutaneously 0.2 mL of the FB1-adjuvanted bovine serum albumin (BSA) 6 times at 14-day intervals. The immunogen complex contained a total of 1 mg of for each immunization prepared with Freund’s adjuvant in equal volume (complete Freund’s adjuvant was used only once; subsequent immunizations were done using incomplete Freund’s adjuvant). Blood was taken before the first injection, and two week later, after the sixth injection. ELISA assay was used to analyze serum samples for FB1-adjuvanted-BSA-specific antibody response. Peripheral blood mononuclear cells (PBMCs) were isolated from blood samples using standardized density gradient technique (Ficoll–Paque) (Solarbio, Beijing, China).

### 5.2. Construction of VHH Phage-Display Library

Total RNA was extracted from lymphocytes according to the instructions on the RNA extraction kit (QIAGEN, Shanghai, China) and reverse-transcribed into cDNA using Oligo (dT) 20 primers. With two cycles of PCR, the variable domain of heavy chain antibody (VHH) gene was amplified. The *VHH* up-forward gene and *VHH* up-reverse gene were used for the first round and *VHH* down-forward gene and *VHH* down-reverse gene were used for the second amplification reaction (Table 1) and the target fragments were then cloned into the pCANTAB 5 E vector and electrically transferred to TG1-competent cells. After three rounds of acid elutions, the bacteriophages bound to FB1-BSA were enriched successfully. In addition, the positive clones were screened by phage-ELISA and the titer of output phage determination was determined. The library diversity and capacity were also identified.

### 5.3. Prokaryotic Expression and Identification of FB1 Nanobody Protein

Based on the target gene sequence, the upstream and downstream primers were designed using Primer 5.0 software (Table 2). Using the above elutriation, the target sequence of *VHH*-FB1-5 was amplified by the *VHH* up-forward gene and *VHH* up-reverse gene, then cloned into a pCold-SUMO vector at Bam HI and Hind III restriction sites (Solarbio, Beijing, China) and identified by bacterial liquid PCR. Positive plasmids were extracted by using OMEGA Plasmid DNA Kit (Omega Bio-tek, Shanghai, China), and the concentration was measured by NanoDrop 2000 (Thermo Fisher Scientific, Waltham, MA, USA) and then sent for sequencing (Sangong Biotech, Shanghai, China) and stored at −20 °C for further use. The *VHH*-FB1-PSF-positive plasmid was transferred to *E. coli* BL21 (DE3) competent cells and induced using 1 M of isopropyl-β-D-thiogalactopyranoside (IPTG, Merck, Beijing, China) at 25 °C for 5 h. The bacterial precipitate was collected by centrifugation and re-suspended with PBS. After ultrasound, the supernatant and precipitate were collected, and SDS-PAGE was used to determine the solubility of the protein. Nanobody 5 (termed Nb5), Nanobody11 (termed Nb11), Nanobody 12 (termed Nb12), Nanobody (termed Nb13) and Nanobody (termed Nb16) were purified individually by Ni-NTA (GenScript, Nanjing, China). Further, the titer of the nanobodies was determined by an indirect ELISA assay. The OD measurement was conducted at OD450 nm.

### 5.4. Detoxification of Nb5 on FB1 in Milk

Milk samples were purchased from the local shop and extracted according to the method previously described [24]. In brief, a skimming process was carried out by centrifugation (4500× *g* for 10 min at 4 °C), and the 9 milk samples were thoroughly vortex mixed. Afterwards, an aliquot of 2 mL of each sample was added to 6 mL of acetonitrile (Aladdin Biochemical Technology Co., Ltd., Shanghai, China), mixed by vortexing for 2 min and processed with ultrasounds for 5 min. The samples were centrifuged to remove large biomolecules (such as proteins) at 12,000× *g* for 15 min. The supernatants were then filtered through 0.22 µm cellulose syringe filters in amber vials until the further HPLC analysis.

Afterwards, FB1 was added to 900 μL of milk to the final concentration of 47.66 ppb and then incubated with 100 μL of 4 dilutions (1:2000, 1:3000, 1:5000, and 1:10,000 of Nb5) at 25 °C for 2 h. Subsequently, the samples were extracted according to the aforementioned method.

### 5.5. Antagonistic Effect of FB1-Specific Nanobody against the Chicken Embryotoxicity of FB1

To analyze the antagonistic effect of nanobodies on FB1 detoxification of embryonated chickens, 64 μg of FB1 was treated separately with a different FB1-specific nanobody (2 μg/egg) at 25 °C, or 0.1 M D-glucose at 70 °C for 2 h while PBS, FB1, and Nanobody were regarded as the control groups.

A total of 48 SPF embryonated chickens, aged 11 days, were purchased from a commercial company (Boehringer Ingelheim Inc, Beijing, China) and divided into 8 groups, 6 embryos per group. The experimental protocols were approved by an Ethical Reviewing Board at the China Agricultural University (approved code: IACUC 20190802, date of approval 2 August 2019), based on guidelines from the Institutional Animal Care and Use Committee of China Agricultural University (IACUC). This follows humane protocols that minimize pain in the animals. The experimental groups were inoculated into albumen with 100 μL of the treated mixtures, and then hatched at 37 °C until day 21 (Table 3). Upon hatching day, chickens were euthanized in a CO_2_ chamber using 100% CO_2_ at a flow rate of 10–30% of the chamber volume per minute, and the birds were observed for the absence of breathing activities and loss of the heartbeat. The CO_2_ flow lasted for at least 1 min after breathing arrest. The body weight and lesions of the lungs and gizzards were observed and scored. The FB1 concentrations from the lungs and gizzards were determined by HPLC (SPD-M20A, Shimadzu Corporation, Japan) as previously described [24].

Forty-eight embryonated eggs aged 11 days were randomly divided into 8 groups, including 5 experimental groups (Nb5, Nb13, BiNb13, BiNb11, and D-glucose groups, 3 replicates per group) and 3 control groups (PBS control, FB1 control, and Nb control, 6 chicken embryos per group). Before inoculation, poorly growing embryonated eggs were eliminated from the experiment.

### 5.6. Antagonistic Effect of Nanobody on Growth, Gizzard Ulceration, and Immune Response of Broilers

A total of 50 ten-day-old Elvin broilers were purchased from a commercial company (Meikeduo Food Group Co. Ltd., Hebei, China). The broilers were randomly divided into 5 groups: 2 experimental groups (Nb5 group and BiNb11 group) and 3 control groups (Control group, FB1 group, and Qinankang + FB1 group) (Table 4). The experimental protocols were approved by an Ethical Reviewing Board at China Agricultural University (Approved code: IACUC 20190803). Qinankang is comprised of 20% protocatechuic acid and reported to degrade 71.43% FB1 at 80 °C for 2 h [26]. It is registered as a commercial detoxifier (Genten Biotech, Beijing, China) against FB1 toxin in poultry. From day 10 onwards, the control group was fed with basic diet while the other groups were given feed with corn gluten meal naturally contaminated with 10 mg FB1 per kg. Simultaneously, broilers orally received 50 μg of Nb5, or 50 μg of BiNb11 and 75 mg/kg of commercial product of Qiangankang for 21 days, respectively. Before treatment, all the birds received an oral dose of attenuated vaccines against NDV and IBV on day 10, as well as one dose of live vaccine against IBDV on day 14. During observation, broilers were weighed weekly and monitored activities. Sera were collected on days 0, 14, and 21. Subsequently, the specific antibody levels for IBV or IBDV, and NDV were determined using commercial ELISA kits (IDEXX Laboratories. Inc, Beijing, China). In addition, lymphocyte proliferation index was determined on day 21 as previously described [33]. At the end of the experiment, the chickens were euthanized humanely using cervical dislocation, which caused death by breaking the blood vessels so that the brains run out of oxygen. Afterwards, gizzard lesions were determined while immune organs were observed, including spleen, thymus, and bursa of Fabricius.

Fifty broilers were randomly divided into 5 groups, including two experimental (Nb5 and BiNb11 groups, ten birds per group) and 3 control groups (Control group, FB1 group, and Qinankang + FB1 group, 10 birds per group). Except for the control group, the other 4 groups received corn gluten meal containing 10 ppm of FB1 per day, and then treated with 50 μg of Nb5, 50 μg of BiNb11, and 75 mg/kg of commercial detoxifier, Qinankang, respectively for 21 days.

As the Table 4 showed, the hatchability was 83.33% and 80%, respectively, in the Nb5 + FB1 group and the D-glucose + FB1 group compared to 33.3% in the FB1 group. Moreover, decreasing hatchability was observed in the Nb13 + FB1 group, the BiNb11 + FB1 group, and the BiNb13 + FB1 group compared to the Nb5 + FB1 group. As for body weight, the FB1 group induced slow growth compared to the control groups and the Nb control group (*p* < 0.01). No statistical difference was found among the Nb5 + FB1 group, or the BiNb11 + FB1 group, the BiNb13 + FB1 group and the D-glucose + FB1 group. Postmortem, FB1 induced a highly gastric ulceration index while lower lesions were determined in the Nb5 + FB1 group and all the control groups. No statistical difference was found among the Nb13 + FB1 group, the BiNb11 + FB1 group, the BiNb13 + FB1 group, and the D-glucose + FB1 group.

As for FB1 residuals, lung and gizzard were collected aseptically, the organs were homogenized, and 1 g of tissue was blended with 5 mL of 80% methanol aqueous solution containing 0.1% acetic acid. Subsequently, the supernatant solutions were filtered by glass fiber filter paper, collected and mixed with 5 mL n-hexane. Afterwards, the samples were centrifugated to discard n-hexane at 12,000 rpm for 5 min. Finally, the samples were collected and diluted with PBS 5 times and passed in column purification. The FB1 residuals were analyzed using high-performance liquid chromatography (Agilent Technologies Inc., Santa Clara, CA, USA). A ZORBAX SB-C18 Column (150 mm × 4.6 mm) was employed with a mobile phase of methanol: sodium biphosphate (77:23). The sample was detected at a flow rate of 1 mL/minute using a 335 nm/440 nm wavelength.

### 5.7. Data Analysis

FB1 concentrations and lesion scores were statistically analyzed using SPSS 17.0 version to perform a one-way ANOVA with the LSD post hoc test on at least three independent replicates. *p*-values of <0.05 were considered statistically significant for each test, and when *p* < 0.01, the results were highly significant. The hatching rate were statistically analyzed using SPSS 17.0 version to perform a Chi-square test with a categorical variable. A *p*-value of <0.05 was considered to be a significant difference for each test and a *p*-value of <0.01 was considered to be highly significant.

## Figures and Tables

**Table 1:** Primers for amplifying *VHH* genes.

Primers	Sequences (5′→3′)
VHH-up-F	GTCCTGGCTGCTCTTCTACAAGG
VHH-up-R	GGTACGTGCTGTTGAACTGTTCC
VHH-down-F	TTTCTATTACTAGGCCCAGCCGGCCATGGCTCAGGTGTGGCTCGTGGAGTC
VHH-down-R	AAGGAAAAAAGCGGCCGCGCCATAATGGCCTGGTTGTG
pCANTAB5-R1	CCATGATTACGCCAAGCTTTGGAGCC
pCANTAB5-R2	CGATCTAAAGTTTTGTCGTCTTTCC

**Table 2:** Primer sequences for constructing recombinant FB1 nanobody prokaryotic expression vector.

Primers	Sequences (5′→3′)
VHH-up-F	CGAGCTCATGGAGGTGCAGCTCCTGGTG
VHH-up-R	CGGGATCCCGAGACGGTGACCAGGGTC

**Table 3:** Experimental protocol of detoxification in the embryonated eggs.

Groups	Treatment	Incubation Conditions	Eggs
Control	PBS	25 °C; 2 h	6
FB1	FB1 (64 µg)	25 °C; 2 h	6
Nb control	1: 50 Nb	25 °C; 2 h	6
Nb5 + FB1	FB1 (64 µg)) + 1: 50 Nb5	25 °C; 2 h	6
Nb13 + FB1	FB1 (64 µg) + 1: 50 Nb13	25 °C; 2 h	6
BiNb11 + FB1	FB1 (64 µg) + 1: 50 BiNb11	25 °C; 2 h	6
BiNb13 + FB1	FB1 (64 µg) + 1: 50 BiNb13	25 °C; 2 h	6
D-glucose + FB1	FB1(64 µg) + 0.1 M D-glucose	70 °C; 2 h	6

**Table 4:** Effect of monomer nanobodies and bivalent nanobodies on hatchability and development of chicken embryos.

Groups	Hatchability (%)	Body Weight (g)
Control	83.33	40.11 ± 1.29 ^a^
FB1	33.33	28.14 ± 7.70 ^b^
Nb5 + FB1	83.33	37.10 ± 2.74 ^a^
Nb13 + FB1	50	32.87 ± 5.47 ^b^
BiNb11 + FB1	60	36.69 ± 3.17 ^a^
BiNb13 +FB1	50	33.61 ± 10.22 ^a^
D-glucose + FB1	80	34.74 ± 5.27 ^a^
Nb control	100	38.35 ± 2.11 ^a^

a indicated *p* > 0.05 when compared the body weight of the Nb5 + FB1 group, the BiNb11 + FB1 group, the BiNb13 +FB1 group, the D-glucose + FB1 group and the Nb control group with that of the control group. b indicated *p* < 0.01 when compared the body weight of the FB1 group and the Nb13 + FB1 group with that of the control group. The data are expressed as the mean ± SD.

## Data Availability

The data presented in this study are available on request from the corresponding author via email address: hecheng@cau.edu.cn.
